# Camelid VHH Antibodies that Neutralize Botulinum Neurotoxin Serotype E Intoxication or Protease Function

**DOI:** 10.3390/toxins12100611

**Published:** 2020-09-24

**Authors:** Jacqueline M. Tremblay, Edwin Vazquez-Cintron, Kwok-Ho Lam, Jean Mukherjee, Daniela Bedenice, Celinia A. Ondeck, Matthieu T. Conroy, Skylar M. L. Bodt, Brittany M. Winner, Robert P. Webb, Konstantin Ichtchenko, Rongsheng Jin, Patrick M. McNutt, Charles B. Shoemaker

**Affiliations:** 1 Department of Infectious Disease and Global Health, Cummings School of Veterinary Medicine, Tufts University, North Grafton, MA 01536, USA; jacqueline.tremblay@tufts.edu (J.M.T.); jean.mukherjee@gmail.com (J.M.); 2 The United States Army Medical Research Institute of Chemical Defense, Fort Detrick, MD 21010, USA; ejv.cintron@gmail.com (E.V.-C.); condeck@genevausa.org (C.A.O.); matthieu.t.conroy.ctr@mail.mil (M.T.C.); smlbodt@gmail.com (S.M.L.B.); winnerbr@msu.edu (B.M.W.); patrick.m.mcnutt2.mil@mail.mil (P.M.M.); 3 Department of Physiology & Biophysics, University of California, Irvine, CA 92697-4560, USA; kwokhl@uci.edu (K.-H.L.); r.jin@uci.edu (R.J.); 4 Department of Clinical Sciences, Cummings School of Veterinary Medicine, Tufts University, North Grafton, MA 01536, USA; daniela.bedenice@tufts.edu; 5 Bacteriology Division, U.S. Army Medical Research Institute of Infectious Diseases, Ft. Detrick, MD 21702-5011, USA; rpwebb2406@gmail.com; 6 Department of Biochemistry and Molecular Pharmacology, New York University School of Medicine, New York, NY 10016, USA; Konstantin.Ichtchenko@nyulangone.org

**Keywords:** botulinum neurotoxin, botulism, toxin, antitoxin, single-domain antibody, VHH, neutralization, protease

## Abstract

Botulinum neurotoxin (BoNT) serotype E is one of three serotypes that cause the preponderance of human botulism cases and is a Tier 1 Select Agent. BoNT/E is unusual among BoNT serotypes for its rapid onset and short duration of intoxication. Here we report two large panels of unique, unrelated camelid single-domain antibodies (VHHs) that were selected for their ability to bind to BoNT/E holotoxin and/or to the BoNT/E light chain protease domain (LC/E). The 19 VHHs which bind to BoNT/E were characterized for their subunit specificity and 8 VHHs displayed the ability to neutralize BoNT/E intoxication of neurons. Heterodimer antitoxins consisting of two BoNT/E-neutralizing VHHs, including one heterodimer designed using structural information for simultaneous binding, were shown to protect mice against co-administered toxin challenges of up to 500 MIPLD_50_. The 22 unique VHHs which bind to LC/E were characterized for their binding properties and 9 displayed the ability to inhibit LC/E protease activity. Surprisingly, VHHs selected on plastic-coated LC/E were virtually unable to recognize soluble or captured LC/E while VHHs selected on captured LC/E were poorly able to recognize LC/E coated to a plastic surface. This panel of anti-LC/E VHHs offer insight into BoNT/E function, and some may have value as components of therapeutic antidotes that reverse paralysis following BoNT/E exposures.

## 1. Introduction

Botulinum neurotoxins (BoNTs) are considered among the most dangerous bioweapon threats (Tier 1 agent, USA Federal Select Agent Program) as a consequence of their extreme toxicity, widespread availability and relative ease of production. BoNTs are also the cause of natural botulism from food poisoning or wound infections. While at least eight different BoNT serotypes are known to exist in nature, serotypes A, B and E are responsible for the vast majority of human cases of botulism [1]. Treatments for exposure to BoNTs are currently limited to antiserum-derived products consisting of purified anti-BoNT polyclonal antibodies (BabyBIG) [2] or F(ab’)_2_ immunoglobulin fragments derived from equine plasma (HBAT) [3]. Pools of humanized anti-BoNT monoclonal antibodies (mAbs) are being developed to replace the antiserum-based products [4,5]. These new and highly effective therapeutics consist of pools of three different mAbs for each BoNT serotype, a factor which complicates their manufacture and increases their cost of production. There is no current antidote for patients in which BoNT has already entered motor neurons and begun to cause flaccid paralysis. Some success has been reported in reversing botulism paralysis with the voltage-gated potassium channel inhibitor 3,4-diaminopyridine (3,4-DAP) [6], and atoxic BoNT vehicles are being developed to deliver biomolecular inhibitors of BoNT proteases to intoxicated neurons [7].

Several research teams have developed camelid single-domain antibodies (called VHHs or nanobodies) as components of antitoxin and antidote products (reviewed by [8,9]). These simple binding agents, which contain the V_H_ region of heavy chain-only antibodies, are small (~14 kDa) proteins that are stable to a wider range of pH and temperatures than mAbs and can be produced inexpensively in microbial hosts [10]. Importantly, VHHs can be expressed as multimers, which impart improved antitoxin potencies [11,12,13] and permit the targeting of multiple toxins or natural toxin variants using a single protein [14,15,16,17,18]. VHH serum persistence can be extended by the addition of an albumin-binding peptide [12] and their in vivo efficacy can be significantly enhanced by co-administering epitopically tagged VHH agents with an anti-tag mAb that promotes toxin clearance [12,19]. VHHs are amenable to genetic delivery and can be expressed for extended times in patients by gene therapy with viral vectors [12] or encapsulated synthetic mRNA [20].

We and others have reported the identification of VHH-based agents that either neutralize BoNT/A or BoNT/B intoxication of cells or inhibit light chain protease domains within the neuronal cytosol [21,22,23,24,25]. Bakherad et al. [26] reported a VHH that neutralized BoNT/E and partially protected against intoxication in mice. VHH-based neutralizing agents (VNAs) consisting of two linked neutralizing VHH components have proven highly effective in protecting mice from BoNT/A and BoNT/B intoxication when administered as proteins [21,25], on red blood cells [27], or by gene therapy [12,20], offering novel therapeutic options for botulism.

Here, we report the characterization of two panels of 37 unrelated, unique VHHs that bind to BoNT/E holotoxin and/or the isolated BoNT/E light chain protease domain (LC/E). VHHs were characterized for the ability to neutralize BoNT/E intoxication of neurons and/or to inhibit LC/E protease cleavage of Synaptosomal-Associated Protein, 25kDa (SNAP-25). To test their in vivo potential as antitoxins, several VHH heterodimeric VNAs were prepared and compared for their BoNT/E antitoxin potencies in mice, including one ‘designer’ VNA consisting of two closely apposed VHHs linked for simultaneous binding to BoNT/E based on crystal structure data [28].

## 2. Results

### 2.1. Discovery and Characterization of BoNT/E and LC/E Binding VHHs

#### 2.1.1. Selection of VHHs Binding ciBoNTE or LC/E Adsorbed to Plastic

**Figure 1:**
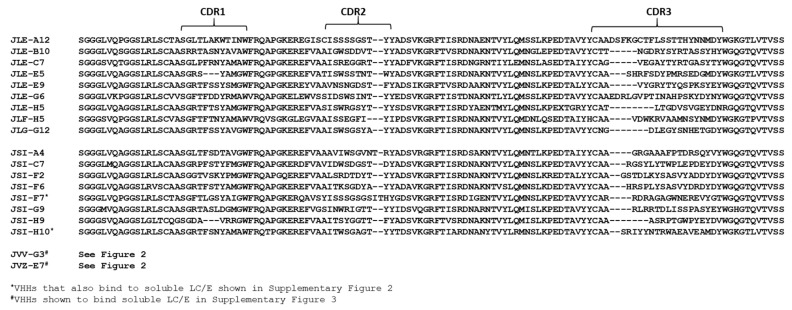
Sequence alignment of all catalytically inactive botulinum neurotoxin serotype E (ciBoNTE)-binding camelid single-domain antibodies (VHHs) identified in this report. Amino acid sequences of all VHHs identified in this report that show clear binding to ciBoNTE on ELISAs employing plastic coated and/or antibody-captured ciBoNTE. Sequences are aligned to conserved framework regions and complementarity-determining regions (CDRs) are indicated.

Two pairs of alpacas (*Vicugna pacos*) were immunized with catalytically inactive BoNT/E (ciBoNTE) [29] and/or LC/E (both subtype E1) as described in the Materials and Methods. VHH display phage libraries were prepared from lymphocyte RNA obtained from each pair of alpacas. Phage displaying VHHs from both libraries were initially selected for binding to ciBoNTE or LC/E proteins directly coated (adsorbed) on high protein-binding Nunc Maxisorp Immuno Tubes. Sequence analysis of VHHs recovered from selection on coated ciBoNTE or LC/E (Figure 1) identified nine VHH families, each comprised of VHHs having distinct homologies among the three complementarity-determining regions (CDRs). When VHH selection was performed on coated LC/E, eleven different VHHs were identified, however only one, JLF-H5, represented a unique family. The remaining VHHs were all members of the JLG-G12 family that was selected on plastic-adsorbed ciBoNTE (Figure 1). One representative of each of the nine VHH families (Table 1 and Table 2) was chosen for expression, purification and characterization.

Competition studies performed among the nine unique ciBoNTE-binding VHHs identified a total of four non-overlapping epitopes (competition groups 1 to 4, Table 1). One epitope on ciBoNTE was recognized by the LC/E-binding VHHs, JLG-G12 and JLF-H5, while the other three epitopes were recognized by the seven remaining VHHs. When assessed by dilution ELISAs performed with ciBoNTE coated on high protein-binding plastic, the different ciBoNTE-binding VHHs displayed unusual variations in peak target binding at high VHH concentrations (Figure S1). In an attempt to explain this observation, we repeated the dilution ELISAs after adsorbing ciBoNTE onto a more hydrophilic tissue culture plastic (Costar) [30]. Surprisingly, VHHs displayed dramatically different binding properties depending on the type of plastic used. For example, the two unique VHHs recognizing the LC/E domain (JLF-H5 and JLG-G12) both displayed much weaker EC_50_ values and reduced levels of target binding on tissue culture vs. protein-binding plastic, suggesting that these VHHs recognize an epitope that is poorly represented in the soluble form of LC/E and becomes much more accessible when the target is coated onto a more hydrophobic plastic surface (Figure S1). This hypothesis was confirmed when we were able to use an antibody to ‘capture’ soluble LC/E and ELISAs found that JLF-H5 and JLG-G12 also displayed minimal LC/E recognition (Figure S1). In contrast, the remaining seven VHHs generally recognized ciBoNTE on tissue culture plastic much better than on protein-binding plastic, particularly in the percent of target binding (Figure S1). These VHHs were also tested by ELISA for recognition of the amino or carboxyl terminal domains of the BoNT/E heavy chain (H_N_E and H_C_E; Table 1 and Table 2) and only two VHHs bound to either protein. The remaining five VHHs therefore recognize multi-domain epitopes of BoNT/E. In total, the ELISA results suggested that ciBoNTE-binding VHHs recognize conformational epitopes that become profoundly altered when ciBoNTE is coated onto different plastic surfaces.

#### 2.1.2. Selection of VHHs Recognizing Antibody-Captured ciBoNTE

Given data that ciBoNTE conformation was substantially changed when coated onto Maxisorp plastic, we repeated the selection process employing ciBoNTE that was captured from solution by an antibody. Specifically, the second round of VHH discovery was performed by panning the VHH display phage library on ciBoNTE had been captured using VHH, JLE-E5. Using the capture method of selection, we identified eight new unique VHH families (JSI series, Table 2, Figure S2) in addition to re-discovering several of the VHHs identified in the prior screens. Seven of the new series of VHHs strongly recognized VHH-captured ciBoNTE on ELISAs with high affinity (EC_50_ ≤ 1 nM). Two of these VHHs (JSI-F7 and JSI-H10) also recognize the VHH-captured form of LC/E (see below).

Competition studies on the JSI series of VHHs indicated that all recognized epitopes that did not overlap with epitopes bound by the VHHs in Table 1 and comprised six new competition groups (Table 2). Two additional ciBoNTE-binding VHHs were identified later among VHHs selected for binding to LC/E (see below). These VHHs (JVV-G3, JVZ-E7) recognize a new epitope on ciBoNTE and their characterization information is included in Table 2. The sequences of all VHHs representing the unique, ciBoNTE-binding VHH families identified through our various panning strategies are included in Figure 1.

#### 2.1.3. Selection of VHHs Recognizing VHH-Captured LC/E

**Figure 2:**
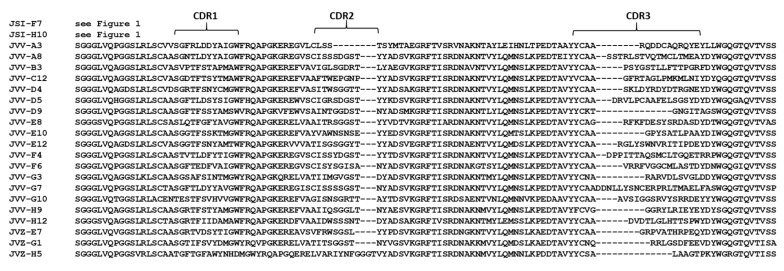
Sequence alignment of all LC/E-binding VHHs identified in this report. Amino acid sequences of all VHHs identified in this report that show clear binding to LC/E on ELISAs employing captured LC/E. Sequences are aligned to conserved framework regions and CDRs are indicated.

Initial screening efforts using LC/E coated to plastic (above) discovered two VHH families that formed a single competition group, represented by JLG-G12 and JLF-H5. Notably, both of these VHHs exhibited poor binding to captured, soluble LC/E. Hypothesizing that adsorption of LC/E to the protein-binding plastic disrupts and unfolds the native, soluble LC/E structure, we re-panned VHH display phage libraries using LC/E captured to a surface with VHH JSI-F7 (Table 2). By re-panning against captured LC/E, we discovered twenty new, unique VHH families (Figure 2). Remarkably, these VHHs showed virtually no recognition of LC/E coated on protein-binding or tissue culture plastic in dilution ELISAs (Figure S3; Table 3). However, these VHHs displayed strong binding to the soluble, VHH-captured LC/E, including 19 VHHs showing EC_50_ ≤ 1 nM. The extreme variations in VHH binding between plastic-coated and captured LC/E further supports the hypothesis of major conformation differences between captured and adsorbed targets.

Given the high affinity of VHHs selected by captured LC/E, we hypothesized a subset of these VHHs would inhibit LC/E protease function. Indeed, nine VHHs clearly inhibited LC/E protease cleavage of a recombinant SNAP-25 following a 1.5 h incubation (Figure S4A; Table 3). Several of these VHHs (e.g., JVV-A3, JVV-B3 and JVV-G7) displayed unusual persistence by maintaining strong LC/E protease inhibition after an 18-h incubation time (Figure S4B). The full set of LC/E-binding VHHs were also tested for protease inhibition in a FRET-based SNAP-25 cleavage assay, Figure S4C. Using this assay, seven of the nine previously identified LC/E protease-inhibitory VHHs reproducibly inhibited SNAP25 cleavage by LC/E, while the remaining two VHHs displayed variable inhibitory activity (Table 3).

### 2.2. VHH and VNA Neutralization of BoNT/E Intoxication

#### 2.2.1. VHH Monomer Neutralization of BoNT/E in Neuronal Cultures

**Figure 3:**
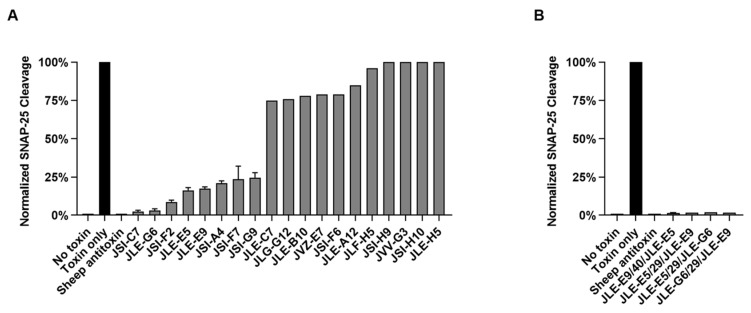
Cell-based assays of all VHH monomers for BoNT/E neutralization. E18 primary rat cortical neurons (17–21 d in culture) were co-incubated with 200 nM VHH or VHH-based neutralizing agents (VNAs) and 16.7 pM BoNT/E (corresponding to 20 MIPLD_50_/mL) for 24 h prior to harvest and immunoblot analysis. SNAP-25 cleavage levels were normalized to toxin-only controls within each experiment and averaged among experiments for VHHs that showed efficacy. (**A**) Summary of cell-based SNAP-25 cleavage assays for all BoNT/A holotoxin-binding VHHs and control treatments. (**B**) Summary of SNAP-25 cleavage assays for all heterodimer VNAs and control treatments.

All unique VHHs that displayed clear binding to ciBoNTE by ELISA (Table 1 and Table 2) were tested for the ability to prevent BoNT/E intoxication of rat E18 primary neuron cultures between 17-21 days in vitro (DIV; Figure 3A; Figure S5) [31]. VHHs were added at a high stoichiometric ratio (200 nM VHH to 16.8 pM BoNT/E) to increase the sensitivity of detecting neutralization. Following overnight incubation, eight unique VHHs were identified that reduced SNAP-25 cleavage by greater than 75% (Figure 3A). All VHHs recognizing LC/E in Table 3 were also tested and unsurprisingly showed no significant BoNT/E-neutralizing activity (Figure S6). Three BoNT/E-neutralizing VHHs (JLE-E5, JLE-E9 and JLE-G6) were identified among the VHHs selected on coated ciBoNTE (Table 1). In co-crystallization studies, JLE-E5 and JLE-E9 have now been studied for their mechanisms of neutralization and both were shown to neutralize intoxication by inhibiting BoNT/E endosomal membrane association [28]. JLE-G6 binds to the H_C_E receptor binding domain of BoNT/E, and thus likely neutralizes the toxin by inhibiting its ability to bind its neuronal receptors, SV2 and/or gangliosides [25]. Five additional BoNT/E-neutralizing VHHs were identified among JSI series VHHs selected on captured ciBoNTE (JSI-A4, JSI-C7, JSI-F2 and JSI-G9). No experimental data regarding their mechanisms of neutralization are yet available for these VHHs, although, like JLE-G6, JSI-F2 binds to the H_C_E receptor binding domain.

#### 2.2.2. VHH Monomer Neutralization of BoNT/E in Mice

We next tested the eight BoNT/E-neutralizing monomer VHHs (2 µg given intravenously) for their ability to protect mice from a co-administered challenge dose of 3 MIPLD_50_ BoNT/E (Figure S7A). Three VHHs were protective under these conditions: JSI-C7, JSI-F7 and JSI-G9, each of which were identified using captured ciBoNT/E. None were protective against a 10 MIPLD_50_ BoNT/E challenge (Figure S7B).

#### 2.2.3. VHH Heterodimer Neutralization of BoNT/E in Mice

Previous studies have demonstrated that expression of VHH heterodimeric VNAs composed of two BoNT-neutralizing VHHs can radically improve in vivo antitoxin potencies [21,25]. To similarly increase the potency of BoNT/E VHHs, we initially engineered and produced VHH heterodimer VNAs using the three BoNT/E-neutralizing VHHs identified in the first screen: JLE-E5, JLE-E9 and JLE-G6. The VNAs were expressed in three different heterodimer combinations, each with the VHH components separated by a 29 amino acid spacer: JLE-E5/29/JLE-E9; JLE-E5/29/JLE-G6 and JLE-G6/29/JLE-E9.

**Figure 4:**
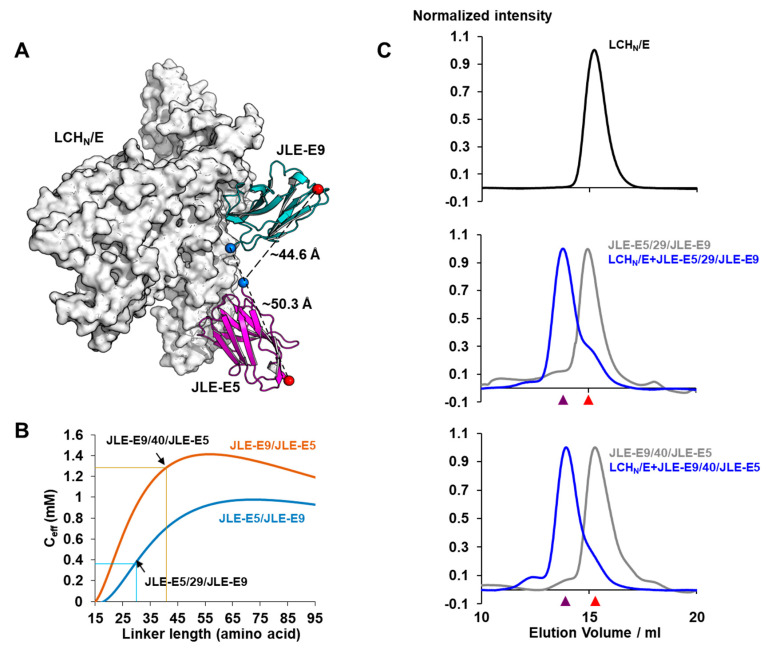
Structure-based design of a VNA optimized for simultaneous binding. (**A**) Inter-molecular distances between JLE-E5 and JLE-E9. The Cα atoms of the N- and C-terminal residues are shown in blue and red spheres, respectively. (**B**) Calculated effective concentration (C_eff_) of tethering JLE-E5 and JLE-E9 in opposite orientations by various linker lengths. The C_eff_ is calculated based on the inter-molecular distances between JLE-E5 and JLE-E9 (a) and according to equation reported in Zhou, 2003 [32], assuming the persistence length of 4.5 Å and the nearest Cα–Cα distance of 3.8 Å. (**C**) Gel filtration analysis. Elution profiles were shown for the LCH_N_/E alone (top panel), or in complex with JLE-E5/29/JLE-E9 (middle panel), or JLE-E9/40/JLE-E5 (bottom panel). The toxin fragment was incubated with VNAs at 2:1 (blue curve) molar ratio. The elution profiles of the free VNAs are colored gray. The peak elution volumes of the monomeric LCH_N_/E–VNA complex and the VNA alone are indicated by purple and red arrows, respectively.

We next produced a fourth ‘designer’ VNA by incorporating new structural information [28] predicted to result in enhanced neutralization properties [25]. Prior studies showed that creating bivalent VNAs that could bind simultaneously to the target is an efficient strategy to increase affinity and potency. The binding of the first VHH is an intermolecular event with the affinity comparable to that of the monovalent VHH, while the second VHH will bind via an intramolecular interaction. The effective concentration (C_eff_) is increased for the second VHH once the first VHH is bound to substantially increase the affinity of the bivalent VNA compared with the individual VHH components. The C_eff_ depends on several factors including the end-to-end distance of the two VHHs, which can be estimated using structural information [32], and the properties and length of the peptide linker. Based on the precise binding sites for JLE-E5 and JLE-E9, which bind to closely apposed epitopes on BoNT/E [28], we engineered a VNA called JLE-E9/40/JLE-E5 which was predicted to have the optimal VHH orientation and spacer length to facilitate the ability of both VHH components to bind simultaneously to their BoNT/E epitopes (Figure 4A,B). To test for simultaneous binding, we performed gel filtration studies (Figure 4C) as previously reported [25]. Co-incubation of JLE-E9/40/JLE-E5 or JLE-E5/29/JLE-E9 with a two-fold molar excess of BoNT/E produced a single peak upon gel filtration, representing the heterodimeric BoNT/E-VNA complex expected when both VHHs are bound to the same molecule (Figure 4C). The high affinities of these VNAs to BoNT/E exceed the detection limits of in vitro assays and are not useful to experimentally differentiate the binding properties of these two VNAs. However, computer modeling can predict affinities and this analysis estimates that the designer VNA JLE-E9/40/JLE-E5 will have a substantially higher binding affinity compared with JLE-E5/29/JLE-E9 (Figure 4B). Neutralization studies in primary neurons revealed that all four VNAs potently blocked BoNT/E intoxication but could not differentiate the potencies of the different VNAs (Figure 3B).

**Figure 5:**
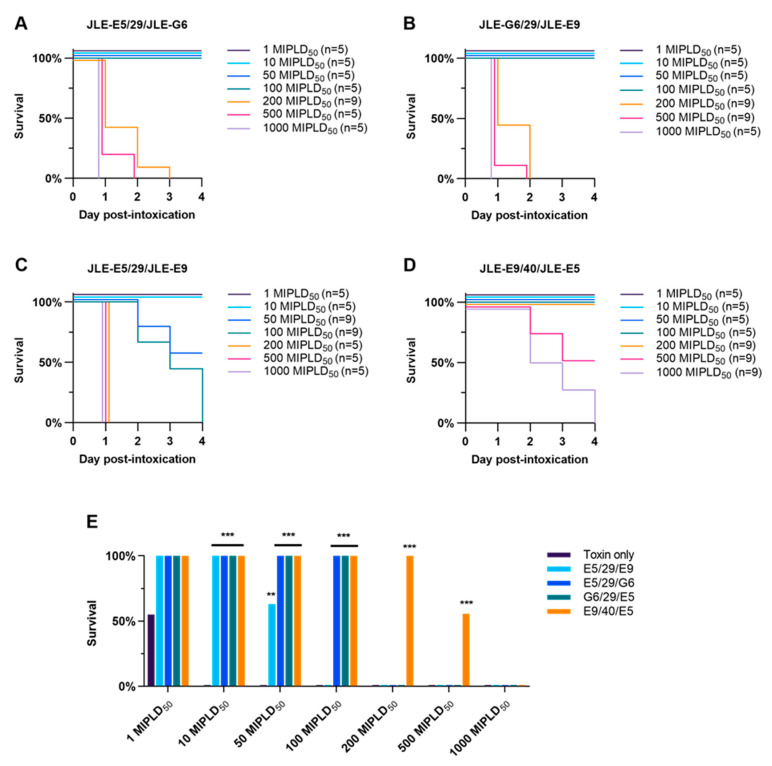
Heterodimer VNAs have radically improved protective efficacies in vivo. Heterodimeric VNAs were prepared containing four different combinations of the three BoNT/E-neutralizing VHHs identified in Table 1 and Table 2. Mice were co-administered 2 µg of the indicated VNA (40 pmol) and 1-1,000 MIPLD_50_ BoNT/E by intravenous injection and survival was monitored over 4 d. (**A**–**D**) Survival curves as a function of time post-exposure for JLE-E5/29/JLE-G6 (**A**), JLE-G6/29/JLE-E9 (**B**), JLE-E5/29/JLE-E9 (**C**) and JLE-E9/40/JLE-E5 (**D**). (**E**) Comparison of protective efficacies among VNAs at each BoNT/E challenge dose, with Bonferroni-adjusted significances shown against vehicle treatments at each dose. *** *p* < 0.001, ** *p* < 0.01.

The ability of VNAs to neutralize BoNT/E was tested in mice by intravenous administration of 40 pmol VNA with 10–1000 LD_50_ of BoNT/E (approximately 0.01–1.0 pmol per mouse). JLE-E5/29/JLE-G6 or JLE-G6/29/JLE-E9 fully protected against 100 LD_50_ BoNT/E challenge but were ineffective in preventing mortality at 200 LD_50_ challenge (Figure 5A,B). Mice treated with JLE-E5/29/JLE-E9 had 63% survival when challenged with 50 LD_50_ of BoNT/E but 0% survival at 100 LD_50_ (Figure 5C). These results were highly consistent with early pilot studies with three of the VNAs performed in a second laboratory employing challenges of 10, 100 and 1000 MIPLD_50_ BoNT/E (Figure S8). In comparison, the designer VNA engineered with optimized orientation and spacer, JLE-E9/40/JLE-E5, fully protected mice co-administered 200 LD_50_ BoNT/E, partially protected mice at 500 LD_50_, and did not protect mice from 1000 LD_50_ (Figure 5D). Optimization of linker length increased neutralizing potency by approximately 10-fold. In addition, the designer VNA was significantly more potent than JLE-E5/29/JLE-G6 and JLE-G6/29/JLE-E9 (Figure 5E). Future studies will prioritize designer VNAs composed of more potent BoNT-neutralizing VHHs from the JSI series once their BoNT/E binding sites are identified and their mechanisms of toxin neutralization are elucidated.

## 3. Discussion

In this paper, we report the discovery of 19 unique, unrelated VHHs recognizing a catalytically inactive form of BoNT/E called ciBoNTE (Table 1 and Table 2). The ciBoNTE employed contains the complete sequence of BoNT/E holotoxin with only a few amino acids modified within the catalytic protease domain to render the toxin effectively inactive [29]. This and other similar catalytically inactive mutant holotoxins appear to retain most or all of the conformational form of the active holotoxins based on their excellent immunogenicity as antitoxins, the presence of a very low level of residual toxicity, and the demonstration that they possess similar receptor binding and crystal structure expected for the active toxin [33,34]. Indeed, we similarly employed catalytically inactivated mutants of BoNT/A and BoNT/B to obtain potent antitoxin VHHs to each serotype [21,25]. Of the 19 unique VHHs recognizing ciBoNTE that we identified, eight were demonstrated to possess significant BoNT/E neutralizing activity.

One major goal of the VHH discovery process targeting BoNT/E is to develop VHH-based antitoxin agents that lead to more effective BoNT antitoxin products. Because of their small size and stable structures, VHHs are highly amenable to multimerization which can dramatically improve the affinity and potency of the products and expand their specificities. This has been demonstrated for a variety of toxin targets including BoNT/A, BoNT/B, Shiga toxins, *C. difficile* toxins, anthrax and ricin [11,13,16,17,18,21,25]. The resulting heteromultimeric VHH-based neutralizing agents (VNAs) alone can be very effective in animal models of intoxication, but where necessary the potency can be further enhanced by co-administering the VNAs with an anti-tag effector antibody (efAb) which binds to two epitopic tags on each VNA to impart the antibody effector functions (e.g., promotion of serum clearance) resident in the Fc domains [16,21,35]. Another approach to enhancing the potency of VNAs is to employ structural information to facilitate the design of VNAs in which two VHH components are both sterically permitted to bind simultaneously to the target. This approach was recently validated as a tool to improve the antitoxin potency of VNAs targeting both BoNT/A and BoNT/B [25].

We report here the use of recent crystal structure and mechanistic data for two BoNT/E-neutralizing VHHs, JLE-E5 and JLE-E9 [28], and the use of these data to predict and then validate the mechanisms by which they neutralize intoxication of neurons. To test these VHHs as heterodimeric VNAs we prepared four different constructs, each containing two of the original three BoNT/E-neutralizing VHHs from Table 1 (JLE-E5, JLE-E9 and JLE-G6). The initial VNAs were designed before structural information became available and contained the three different possible pairs with the three VHHs. The fourth VNA exploited structural information showing that JLE-E5 and JLE-E9 bind nearby on BoNT/E and was designed with the VHH orientation and separating spacer length optimized to permit both VHH components to bind simultaneously on the toxin. As we expected based on our prior studies with other toxins, each of the heterodimers (dosed at 40 pmole/mouse) were capable of protecting mice from co-intoxication with lethal doses of BoNT/E, though having different upper limits on the toxin dose with which they were effective. The initial heterodimer consisting of the two VHHs, JLE-E5 and JLE-E9, which neutralize BoNT/E by the same mechanism (inhibiting H_N_ membrane insertion, proved to be the least potent and protected mice only up to about 50 LD_50_ BoNT/E. The two VNAs containing VHH pairs the neutralize BoNT/E by inhibiting both H_N_ membrane insertion and receptor binding (JLE-G6) fully protected mice from 100 LD_50_ BoNT/E. The fourth VNA in which the orientation and spacer length were optimized to freely permit simultaneous binding of JLE-E5 and JLE-E9 proved to be the most potent, protecting most mice to 500 LD_50_ BoNT/E. Thus the ‘designer VNA’ was about 10× more potent than the other VNA with the same VHH components that lacked optimization for simultaneous binding. We now also report four new unique BoNT/E-neutralizing VHH monomers, and three of these appear to be more potent than the JLE panel VHHs (Table 1 and Table 2) based on mouse studies with low dose toxin challenges. Once structural and mechanistic information is available with these VHHs from the JSI panel (Table 2), we anticipate the ability to design VNAs having substantially improved potencies.

The VHH phage display library from alpacas immunized with ciBoNTE or LC/E was initially panned by selection for phage binding to the two proteins coated onto standard Nunc Maxisorp plastic wells. This plastic offers both hydrophobic and hydrophilic binding sites to maximize adsorption of proteins. While this is an advantage for most proteins, coating to this surface can alter the conformation of some proteins [36]. We noted that all the numerous VHHs we obtained from panning on ciBoNT/E which showed recognition of LC/E fell into two homology groups which competed for the same epitope. A separate panning round on Maxisorp coated LC/E also yielded only similar VHHs in the same competition group. This group of 11 VHHs included 10 related VHHs best represented by JLG-G12, and all showed clear recognition of LC/E coated to protein-binding plastic but were virtually unable to bind to the soluble form of LC/E. Since VHH antibodies are known to be strongly dependent on target conformation for binding [37], we speculated that these VHH bound to a partially unfolded conformation of LC/E. We then re-panned the VHH-display phage library on soluble LC/E that had been captured to a surface with an antibody. The results were dramatically different as we identified a robust panel of 20 unique, unrelated VHHs recognizing native LC/E, none of which were related to the JLG-G12 competition group.

These findings strongly suggested that the LC/E domain of ciBoNTE undergoes a major conformational change upon adsorption to a plastic surface. We do not believe that coating on plastic completely denatures LC/E because several members of the JLG-G12 family of VHHs were tested for recognition of LC/E on denaturing western blots and produced undetectable or very weak signals (not shown). In other studies, we found that ciBoNTE adsorbed to tissue culture plastic became much better recognized by JLG-G12 when the ciBoNTE was denatured by pretreatment with boiling phosphate-buffered saline (PBS) or 0.1M HCl (Figure S9). Similar ciBoNTE treatments resulted, as expected, in near loss of binding by the JLE-E5 VHH which recognizes native ciBoNTE. The results seem more consistent with the hypothesis that LC/E is induced to become a stable, partially unfolded conformation recognized by the JLG-G12 family upon adsorption to plastic, more so on hydrophobic protein-binding plastic than tissue culture plastic. When tissue culture plastic-bound ciBoNTE is transiently denatured by heat or pH, it refolds back into the partially unfolded state. It seems likely that this unusual lability of LC/E is a major reason for the notoriously poor expression and poor solubility of this protease [38]. We hypothesize that this extreme lability of shape upon plastic adsorption reflects an intrinsic rapid ability of LC/E to undergo the transition to a proposed ‘molten globule’ form during association with endosomal membrane while at lower pH undergoing neuronal transcytosis to the cytoplasm [39,40], and this unusual lability compared to other BoNT proteases [41] may be at least one reason why BoNT/E is such a fast-acting poison in animals. Future studies will seek to determine whether JLG-G12 recognizes a specific conformational epitope present in the molten globule state.

Because of the apparent lability of LC/E coated to plastic, we employed a LC/E-binding VHH, JSI-F7 (one of two such VHHs obtained by panning on captured ciBoNTE), as a LC/E capture agent to permit a new panning of our VHH display phage library on native protease. Using native LC/E target for panning, we discovered a robust panel of 20 new unique and unrelated VHHs (JVV and JVZ panels) that recognize epitopes on LC/E (Table 3). None of the VHHs identified in this panning were related to JLG-G12. Two of the new LC/E-binding VHHs, JVV-G3 and JVZ-E7, recognize captured ciBoNTE, both with sub-nM EC_50_. The remaining 18 VHHs in the JVV and JVZ panels must bind LC/E domain epitopes that are partially or completely hidden by the heavy chain within the holotoxin form. Of the 22 different unique LC/E binding VHHs in Table 3, 9 VHHs proved able to inhibit the SNAP-25 protease activity of LC/E. Intraneuronal delivery of LC/E protease inhibitor VHHs via biomolecular vehicles may be a viable approach to development of novel botulism antidotes capable of accelerating recovery from paralysis. Towards this goal, the cytosolic delivery of protein cargo to neurons has recently been demonstrated using heat-labile enterotoxin II [42] and atoxic mutants of BoNT [7,43].

## 4. Conclusions

In summary, we report the discovery and characterization of 19 unique VHHs capable of binding ciBoNTE holotoxin and 22 unique VHHs able to bind to soluble LC/E domain. Seven of the ciBoNTE-binding VHHs significantly inhibited BoNT/E intoxication of neurons. Heterodimers of two linked BoNT/E-neutralizing VHHs were highly protective of BoNT/E intoxication in mice, particularly a heterodimer that was optimized to permit simultaneous binding of both VHH components to the toxin. LC/E-binding VHHs were able to bind only to the protease when it was adsorbed to plastic or when it was immobilized suggesting the protease is highly labile and easily switches between a soluble form and a completely different conformation when associated with a hydrophobic surface. Nine VHHs recognizing soluble LC/E were capable of inhibiting its protease activity. These new VHHs may prove to be useful research reagents and valuable components of novel BoNT/E antitoxin and/or antidote therapeutics.

## 5. Materials and Methods

### 5.1. Ethics Statement and Disclaimers

Animal studies were conducted with the approval of Institutional Animal Care and Use Committees from the U.S. Army Medical Research Institute of Chemical Defense (USAMRICD) and Tufts Cummings School of Veterinary Medicine (TCSVM) conducted under Protocol G2016-74, 7/18/2016. All procedures were conducted in accordance with the principles stated in the Guide for the Care and Use of Laboratory Animals [44], and the Animal Welfare Act of 1966 (P.L. 89–544), as amended. Animal facilities were maintained in accordance with the Animal Welfare Act, United States Department of Agriculture Regulations (9 CFR, Parts 1, 2, and 3), and the Guide for the Care and Use of Laboratory Animals. USAMRICD and the Cummings School have Public Health Service-approved Animal Welfare Assurance Agreements (A4528-01 and A4059-01, respectively) on file with the NIH Office of Laboratory Animal Welfare. The views expressed in this manuscript are those of the authors and do not reflect the official policy of the Department of Army, Department of Defense, or the U.S. Government.

### 5.2. Toxins and Reagents

#### 5.2.1. BoNT/E Toxin and ciBoNTE Immunogen

Purified single-chain botulinum neurotoxin serotype E (BoNT/E; Metabiologics, Inc.) was activated by a 60 min incubation at 37 °C in 0.05 M sodium phosphate buffer (pH 6.5), 0.3 mg/mL TPCK-treated trypsin (Sigma-Aldrich, St Louis, MO, USA) and 10% glycerol [31]. Activated BoNT/E was diluted 1:4 with gelatin (Sigma-Aldrich; St. Louis, MO, USA) and stored at −80 °C until use. The mouse intraperitoneal median lethal dose (MIPLD_50_) of each lot of activated BoNT/E was determined by simple logistical regression using the mouse lethal assay [45]. Prior to injection, BoNT/A was diluted to various working concentrations in PBS with 0.2% gelatin (ThermoFisher, Waltham, MA). Sheep anti-BoNT/E antiserum was produced, and its potency determined, as previously described [21]. The ciBoNTE [33] was kindly provided by Dr. Robert Webb, USAMRIID. Antibodies used were mouse anti-SNAP25 antibody (SMI-81; Sigma); goat anti-rabbit HRP antiserum (Sigma); HRP anti-E-tag mAb (Bethyl).

#### 5.2.2. LC/E Protein

The expression construct for the light chain of BoNT/E1, called LC/E, was designed and synthesized for optimized expression in *E. coli* with tandem affinity tags—8His residues on the N-terminus and 3 streptag II repeats on the C-terminus. Affinity tags were separated from the sequence of the LC/E1 (aa 2–403) by the sequence recognized and cleaved by Tobacco Etch Virus (TEV) protease. The synthetic gene with affinity tags was subcloned into pET28a Kan^+^ expression vector and transformed into BL21(DE3) *E. coli* expression host. Protein expression was performed in auto-induction medium [46]. Briefly, a single colony of *E. coli* was grown overnight in 50 mL of ZYM5052 medium (*Studier*) supplemented with 100 μg/mL kanamycin at 37 °C, 250 rpm, in 125 mL baffled flask (final OD_600_ ~ 7–12). In total, 500 mL of freshly prepared ZYM5052 medium in 2 L baffled flask supplemented with 100 μg/mL kanamycin was inoculated with 1.5 mL of the overnight culture and grown at 37 °C, 300 rpm, until OD_600_ reached ~0.5. Once OD_600_ ~ 0.5 was reached, growing continued for 16 h at 18 °C, 275 rpm. At 16 h cells were harvested by centrifugation (5000 g, 4 °C, 0.5 h) and resuspended in ice-cold Buffer A (500 mM NaCl, 25 mM sodium phosphate, pH 8.0, ~1 g cell paste / 10 mL buffer), supplemented with protease inhibitors (Thermo Fisher Scientific, Cat A32965, 1 tablet for every 50 mL of suspension). The cell suspension was lysed by three passes using Avesin EmulsiFlex C-3 homogenizer (PRC). Triton X-100 was added to the lysate to a final concentration 0.1% and the mixture was kept on a stirring plate for 0.5 h at 4 °C. Subsequently the lysate was cleared by centrifugation (Ti-45 rotor, ~40000 rpm, 0.5 h, 4 °C) and the supernatant was collected. Supernatant was loaded on the Ni^2+^-NTA resin (Qiagen, ~20 mL bed volume), pre-equilibrated with Buffer A supplemented with 0.1% Triton X-100. After loading, the column was washed with 5 volumes of Buffer A supplemented with 0.1% Triton X-100, and subsequently with 5 volumes of Buffer A supplemented with 10 mM and 30 mM imidazole. The fraction containing the enriched LC/E was eluted with 5 volumes of Buffer A (~100 mL) supplemented with 300 mM imidazole, final pH 8.0. Protease inhibitors (1 tablet for 50 mL) and EDTA (to final concentration 5 mM) were added to the Ni^2+^-NTA eluate and the mixture was loaded on the Streptactin high capacity resin (IBA Gmbh, bed volume ~ 20 mL), pre-equilibrated with Buffer A, supplemented with 300 mM imidazole, 5 mM EDTA. After loading, the column was subsequently washed with 10 volumes of Buffer B (1 M NaCl, 50 mM sodium phosphate, 5 mM EDTA, final pH 8.0), 10 volumes of Buffer C (100 mM NaCl, 25 mM sodium phosphate, 10% glycerol, 5 mM EDTA, final pH 8.0) and protein was eluted in 10 volumes (~200 mL) of Buffer C supplemented with 10 mM D-desthiobiotin. The eluate was concentrated ~50 fold using spin concentrators 10K MWCO at ~3000 g. The LC/E was further by size exclusion chromatography on a HiLoad 26/600 Superdex 200 pg column (GE) equilibrated with buffer C. Fractions containing LC/E were consolidated and, concentrated using spin concentrators 10K MWCO at ~3000 g, then diluted 1:1 v/v with Buffer C, 80% glycerol. The protein concentration was determined with Pierce BCA Protein Assay kit (Thermo Fisher Scientific). Protein was aliquoted and stored at −20 °C.

### 5.3. Alpaca Immunization, VHH Discovery and Purification of Recombinant Proteins

Four alpacas were immunized at different times with various antigens representing BoNT/E. Two alpacas were immunized with glutathione-S-transferase fusion proteins to LC/E (kindly provided by Dr. Randall Kincaid). Two alpacas were immunized with a pool of purified recombinant catalytically inactive BoNT/E holotoxin, subtype 1 (ciBoNTE) and recombinant LC/E. All alpacas were immunized by five multi-site subcutaneous (SC) injections at approximate 3-week intervals essentially as previously described [17]. Between 3 to 5 days following the fifth immunization, blood was obtained for lymphocyte preparation. RNA was prepared from lymphocytes using the QIAshredder homogenizer and RNeasy Plus Mini kit (Qiagen, Valencia, CA, USA) as recommended by the manufacturer. Two VHH-display phage libraries were prepared, each from the lymphocyte RNA from pools of two alpacas, essentially as described previously [11]. Two different VHH-display phage libraries were prepared, each having a complexity of >10^7^ independent clones with >95% containing VHH inserts.

The VHH-display phage libraries were panned to enrich and then identify VHHs binding to ciBoNTE or to LC/E using methods essentially as previously described [11,21]. Briefly, following two rounds of panning to a protein, clones expressing VHHs that recognize the protein were identified by ELISA and compared by DNA fingerprinting [16]. VHH coding DNA was obtained for clones with distinct fingerprints and aligned. One VHH representing each unique, clonally related family (i.e., containing clear CDR homology) was selected for soluble expression and purification (see below). Early VHHs were obtained by panning on 1 µg/mL ciBoNTE (JLE, JLF, JLG series) or 1 µg/mL LC/E that had been immobilized on protein-binding plastic. To select for VHHs binding to more conformationally native ciBoNTE, later panning was performed on 1 µg/mL ciBoNTE that had been captured onto Nunc Immuno Tubes previously coated with 5 µg/mL of purified JLE-E5 VHH expressed with a myc tag (JSI series). Similarly, VHHs recognizing native LC/E were selected on 1 µg/mL LC/E that was captured onto plastic previously coated with 5 µg/mL of JSI-F7 expressed with a myc tag (JVV and JVZ series). VHHs that displayed good binding properties for ciBoNTE as purified proteins are shown in Table 1 and Table 2. Unique VHHs showing good binding properties to LC/E are shown in Table 3. The VHH coding DNAs were expressed and purified from *E. coli* as thioredoxin (Trx) fusion proteins with a carboxy-terminal E-tag, or as VHHs lacking Trx with myc tags, as previously described [21]. VHH heterodimers were encoded as synthetic DNA and similarly expressed and purified as Trx fusion proteins. The VHHs in the heterodimers were initially separated by 29 amino acids containing the VHH hinge region and a 15 amino acid spacer (GGGGS)_3_. The optimized heterodimer, JLE-E9/40/JLE-E5, lacked the hinge region and contained a (GGGGS)_8_ spacer.

### 5.4. Dilution ELISAs

ELISAs were performed employing either protein-binding or tissue culture 96-well plates. For capture ELISAs, the capturing agent was typically 5 µg/mL of a VHH expressed with an amino terminal 6His tag and a carboxyl terminal myc tag. Where indicated, 5 µg/mL of streptavidin was coated to capture LC/E that was expressed with a carboxyl terminal strep-tag. For VHH capture ELISAs, we employed VHHs expressed with a myc tag and coated at 5 ug/mL. BoNT/E protein targets were typically coated at 1 ug/mL overnight at 4 °C in PBS. All coated proteins were blocked for at least an hour at 37 °C with 4% milk in PBS, 0.1% Tween. For streptavidin or VHH capture ELISAs, the target (ciBoNTE or LC/E) was incubated at 1 µg/mL for one hour at 37 °C with 4% milk in PBS, 0.1% Tween. After washing, dilution ELISAs were then initiated by diluting the test VHHs (expressed in pET-32 with an amino terminal thioredoxin and a carboxyl terminal E-tag) to 125 nM and performing serial dilutions of 1:5. After incubation for one hour at 37 °C, plates were washed and then incubated with 1:10,000 goat HRP/anti-E-tag (Bethyl) for one hour, washed, developed with TMB (Sigma) as recommended by manufacturer and read at A450.

### 5.5. Competition Assays

Competition assays were performed essentially as previously described [21]. In some cases, in place of phage, we employed soluble, purified VHHs expressed with myc tags, as competitors in ELISA assays detecting target binding by purified VHHs expressed with E-tags.

### 5.6. Cell-Based BoNT/E Neutralization Assays

Prescreening assays to assess the BoNT/E neutralizing activity of the first set of VHHs were performed using methods very similar to those reported previously [47] for BoNT/A except that 2.5 nM BoNT/E was used in the overnight incubations. Cell-based assays for VHH neutralization of BoNT/E were conducted using fresh E18 rat hippocampal, cortical and ventricular zone tissues obtained from BrainBits (Springfield, IL, USA), dissociated according to the manufacturer’s instructions, and plated at a density of 175,000 cells/cm^2^ in 12-well plates coated with polyethylenimine and laminin (Sigma-Aldrich) as previously described [31,48]. Neuronal cultures were maintained at 5% CO_2_, 37 °C, and 95% humidity in NbActiv4 medium (BrainBits). Experiments were performed 17 to 21 days after plating. For neuronal intoxication, 167 pM BoNT/E was pre-mixed with 2 uM of each VHH or VNA in conditioned medium supplemented with 0.2% gelatin (Sigma) for 15 min at 37 °C and added 1:10 to neurons to a final concentration of 16.7 pM BoNT/E (20 MIPLD50/mL) and 200 nM each VHH or VNA. Controls included naïve neurons, sheep anti-BoNT/E antiserum and vehicle treatments. Neurons were lysed after 24 h and protein from neuronal cultures was harvested, quantified by BCA (Pierce), separated by SDS-PAGE and transferred to PVDF. Blots were stained using primary antibodies against SNAP-25 (SMI-81; Covance, Princeton, NJ, USA) and STX1 (110011; Synaptic Systems, Gottingen, Germany) and visualized using goat anti-mouse secondaries conjugated to Alexa Fluor 488 (Invitrogen, Carlsbad, CA, USA) for detection of STX1 (33 kDa) and intact or cleaved SNAP-25 (18 or 25 kDa, respectively). The percentage of cleaved SNAP-25 was determined by densitometry, normalized to toxin-only controls within each experimental and averaged among experiments. Studies were averaged among 2–4 assays for treatments that showed efficacy.

### 5.7. LC/E Protease Inhibition Assay

Initial assays to screen VHHs for their ability to inhibit LC/E protease were performed using the Repcon5 substrate provided by the Ichtchenko lab. This protein consists of rat SNAP-25 aa 128–206 flanked by 12 histidines and mWasabi on the amino side and rat VAMP aa 2–96, 3 copies of streptag and avitag on the carboxyl side. This 56 kDa protein is cleaved into a 36 kDa and 20 kDa product upon cleavage by LC/E. Incubations were at 37 °C performed in 20 mM HEPES pH 8.0, 20 µM zinc acetate and 0.1% Tween-20. Unless otherwise indicated, reactions were 40 µl containing 40 ng of LC/E, 400 ng of purified recombinant VHH protein and 4 µg of Repcon5. After 90 min, a 20 µL aliquot of the reaction were added to 20 µL of 2× SDS sample buffer and placed in a 100 °C heat block for 5 min. SDS PAGE was performed on 10 µl aliquots of the samples. Substrate cleavage was detected by Western blotting with HRP/anti-hexahistidine (Santa Cruz). Assays were repeated on all VHHs at least three times. BoTest^®^ assays were performed basically as recommended by the manufacturer (BioSentinel). For these assays, we employed 25 ng/mL of LC/E and variable amounts of VHHs.

### 5.8. Standard Mouse Toxin Lethality Assay

BoNT/E protection studies were conducted in male CD-1 mice (10–14 weeks of age; Charles River Laboratories, Wilmington, MA (female mice used only in Figure S8)). Mice were group-housed, maintained on a 12 h diurnal cycle and provided a standard diet with regular enrichment and water *ad libitum*. Mice weighing 24–28 g were randomly assigned to groups for all experiments. Intoxication was performed by diluting 1–1000 MIPLD_50_ BoNT/E in a total volume of 200 uL PBS supplemented with 0.2% gelatin (Sigma) and 2 µg (40 pmol) of each VHH or VNA. The mixture was incubated at 37 °C for 15–30 min and administered to mice by tail vein injection. Survival was monitored at 24 h intervals over 4 to 7 days. To minimize animal distress, mice were euthanized if they presented with terminal signs of botulism such as severe agonal breathing and total paralysis, as previously described [49]. Typical study sizes were 3–5 mice per group, repeated 2–3 times. Survival outcomes among 3 or more groups were initially compared using the Chi-square test. If significant differences existed within the experimental group (*p* < 0.05), Fisher’s exact test was used to make pairwise comparisons against vehicle controls. To compensate for multiple hypothesis testing, the Bonferroni-adjusted significance threshold was determined by dividing 0.05 by the total number of pairwise comparisons.

### 5.9. Gel Filtration Analysis

LCH_N_/E (M1–K845) was purified as described [28]. In total, 0.8 µM of LCH_N_/E was incubated with JLE-E5/29/JLE-E9 or JLE-E9/40/JLE-E5 at a LCH_N_/E:VNA = 2:1 molar ratio in PBS at 4 °C for 1 h. The protein mixtures, the individual VNA, or the LCH_N_/E was then separated by Superdex-200 size-exclusion chromatography in PBS. The experiment was performed in duplicate.

## Figures and Tables

**Table 1:** Characterization of ciBoNTE-binding VHHs selected on plastic-adsorbed ciBoNTE or BoNT/E light chain protease domain (LC/E).

VHH Name	ciBoNTE-Binding EC_50_, nM Nunc ^A^	ciBoNTE-Binding EC_50_, nM Costar ^A^	H_N_E-Binding EC_50_, nM Costar ^B^	H_C_E-Binding EC_50_, nM Costar ^B^	LC/E-Binding ^A^ EC_50_, nM Nunc ^A^	BoNT/E Neutralization ^C^	Competition Group ^D^
JLE-A12	1	1	NB	NB	NB	-	1
JLE-B10	3	10	NB	NB	NB	-	1
JLE-C7	3	1	NB	NB	NB	-	1
JLE-E5	1	1	NB	NB	NB	+	2
JLE-E9	1	1	NB	NB	NB	+	1
JLE-G6	1	20	NB	3	NB	+	3
JLE-H5	10	>50	20	NB	NB	-	1
JLF-H5	20	>100	NB	NB	10	-	4
JLG-G12	3	>100	NB	NB	2	-	4

^A^ EC_50_ estimates based on dilution ELISAs (e.g., Figure S1). ^B^ EC_50_ estimates based on dilution ELISAs (not shown). ^C^ BoNT/E neutralization based on assays shown in Figure 3. ^D^ Distinct epitopes based on competition analysis (see Methods). NB—no binding.

**Table 2:** Characterization of ciBoNTE-binding VHHs selected on VHH-captured ciBoNTE or LC/E.

VHH Name	ciBoNTE-Binding EC_50_, nM Captured ^A^	LC/E-Binding EC_50_, nM Captured ^A^	H_N_E-Binding EC_50_, nM Costar ^B^	H_C_E-Binding EC_50_, nM Costar ^B^	BoNT/E Neutralization ^C^	Competition Group ^D^
JSI-A4	10	NB	NB	NB	+	5
JSI-C7	0.8	NB	NB	NB	+	6
JSI-F2	0.5	NB	NB	2	+	7
JSI-F6	1	NB	NB	3	-	8
JSI-F7	0.3	2	NB	NB	+	9
JSI-G9	0.3	NB	5	NB	+	10
JSI-H9	0.6	NB	>100	NB	-	6
JSI-H10	0.2	0.4	NB	NB	-	6
JVV-G3 ^E^	2	0.6	ND	ND	-	11
JVZ-E7 ^E^	3	0.4	ND	ND	-	11

^A^ EC_50_ estimates based on dilution ELISAs with ciBoNTE captured by JLE-E5 or JSI-F7 (Figure S2). ^B^ EC_50_ estimates based on dilution ELISAs (not shown). ^C^ BoNT/E neutralization based on assays shown in Figure 3. ^D^ Distinct epitopes based on competition analysis (see Methods). ^E^ Additional characterization shown in Table 3.

**Table 3:** Characterization of soluble LC/E-binding VHHs.

VHH Name	LC/E-Binding EC_50_ (nM) Nunc ^A^	LC/E-Binding EC_50_ (nM) Costar ^A^	LC/E-Binding EC_50_ (nM) JSI-F7-Captured ^A^	LC/E Protease Inhibition
JSI-F7 ^B^	>25	>25	>25 *	-
JSI-H10 ^B^	>25	>25	0.03	-
JVV-A3	>25	>25	0.4	++
JVV-A8	>25	>25	0.8	++
JVV-B3	>25	10	0.4	++
JVV-C12	>25	10	0.2	-
JVV-D4	>25	>25	0.4	-
JVV-D5	>25	>25	0.2	-
JVV-D9	>25	5	0.2	-
JVV-E8	>25	>25	0.2	-
JVV-E10	>25	>25	0.4	++
JVV-E12	>25	>25	0.8	-
JVV-F4	>25	>25	0.2	-
JVV-F6	>25	2	0.4	-
JVV-G3 ^B^	>25	>25	0.6	+
JVV-G7	>25	>25	0.4	++
JVV-G10	>25	>25	0.4	-
JVV-H9	>25	>25	1	-
JVV-H12	>25	10	0.4	++
JVZ-E7 ^B^	>25	>25	0.4	+
JVZ-G1	>25	>25	2	-
JVZ-H5	>25	>25	0.4	++

^A^ EC_50_ estimates based on dilution ELISAs (e.g., Figure S3). ^B^ Additional characterization in Table 2. ++ VHHs showed inhibition on Repcon5 assay and FRET assays (Figure S4). + VHHs showed inhibition on Repcon5 assay but variable activity on FRET assays (Figure S4). * Binding blocked by the VHH used for target capture.

## Supplementary Materials

The following are available online at https://www.mdpi.com/2072-6651/12/10/611/s1, Figure S1: Dilution ELISAs of all ciBoNTE-binding VHHs tested on plastic coated ciBoNTE and LC/E, Figure S2: Dilution ELISAs of all JSI-series VHHs for captured ciBoNTE or LC/E, Figure S3: Dilution ELISAs of LC/E-binding VHHs on plastic coated or antibody-captured LC/E. Figure S4: Screening of LC/E-binding VHHs for protease inhibition activity, Figure S5: A subset of VHHs and VNAs have neutralizing properties in rat primary neuron cultures, Figure S6: Summary of cell-based SNAP-25 cleavage assays for non-neutralizing VHHs, Figure S7: Select VHH monomers are able to protect mice from co-intoxication with 3 MIPLD50 BoNT/E, Figure S8: Second site study testing the BoNT/E antitoxin potency of three VHH heterodimers in mice, Figure S9: The JLG-G12 VHH family recognizes a stable, partially unfolded ciBoNTE conformation.
