# Supplementary material for: Camelid VHH Antibodies that Neutralize Botulinum Neurotoxin Serotype E Intoxication or Protease Function
Source: Toxins (Basel). 2020 Sep 24;12(10):611. doi: 10.3390/toxins12100611 (PMC7598594; doi:10.3390/toxins12100611)
Supplement: Supplementary file 1 [file toxins-12-00611-s001.pdf]

Toxins **2020**, *12*, 611; doi: 10.3390/toxins12100611

Supplementary Materials: Camelid VHH Antibodies that Neutralize Botulinum Neurotoxin Serotype E Intoxication or Protease Function

Jacqueline M. Tremblay, Edwin Vazquez-Cintron, Kwok-Ho Lam, Jean Mukherjee, Daniela Bedenice, Celinia A. Ondeck, Matthieu T. Conroy, Skylar M. L. Bodt, Brittany M. Winner, Robert P. Webb, Konstantin Ichtchenko, Rongsheng Jin, Patrick M. McNutt and Charles B. Shoemaker

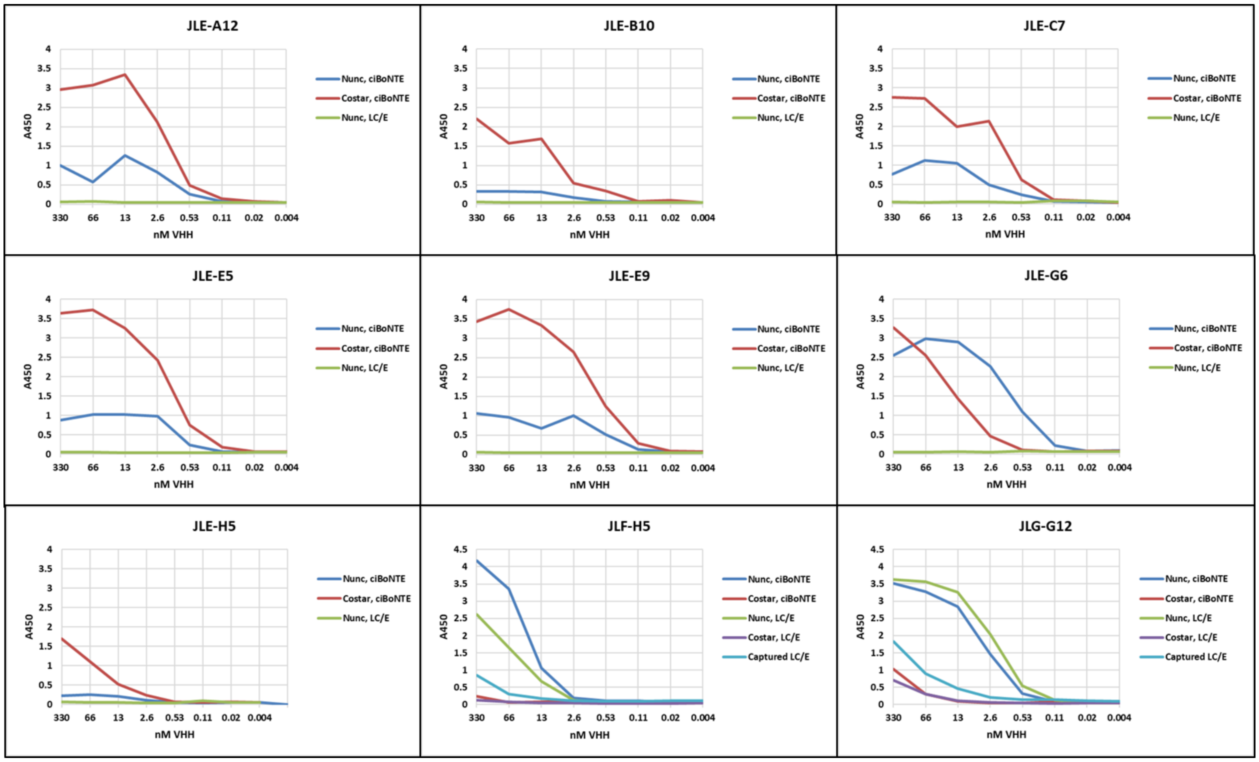

Figure S1. Dilution ELISAs of all ciBoNTE-binding VHHs tested on plastic coated ciBoNTE and LC/E. The ciBoNTE ELISAs were performed by coating 1 µg/mL of the target onto either protein-binding (Nunc) or tissue culture plastic (Costar) wells as indicated. LC/E ELISAs were performed by coating 1 µg/mL of the target onto Nunc Maxisorp plastic. All VHHs were diluted to 125 nM and then subjected to 1:5 serial dilutions. VHH binding was detected with HRP/anti-E-tag reagent.

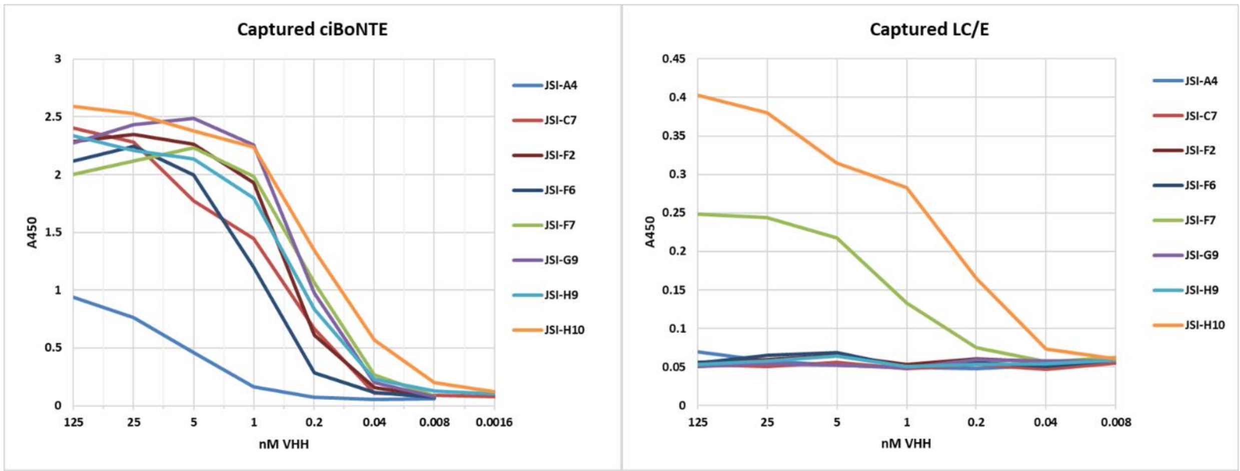

Figure S2. Dilution ELISAs of all JSI-series VHHs for captured ciBoNTE or LC/E. ELISA plates for captured ciBoNTE employed 5 μg/mL of VHH JLE-E5 (expressed with a myc tag) pre-coated onto plates prior to capture of 1 μg/mL ciBoNTE. All VHHs were diluted to 125 nM and then subjected to 1:5 serial dilutions. VHH binding was detected with HRP/anti-E-tag reagent. ELISA plates for captured LC/E employed VHH 5 μg/mL streptavidin pre-coated onto plates prior to capture of 1 μg/mL LC/E (expressed with strep-tag). All VHHs were diluted to 125 nM and then subjected to 1:5 serial dilutions. VHH binding was detected with HRP/anti-E-tag reagent.

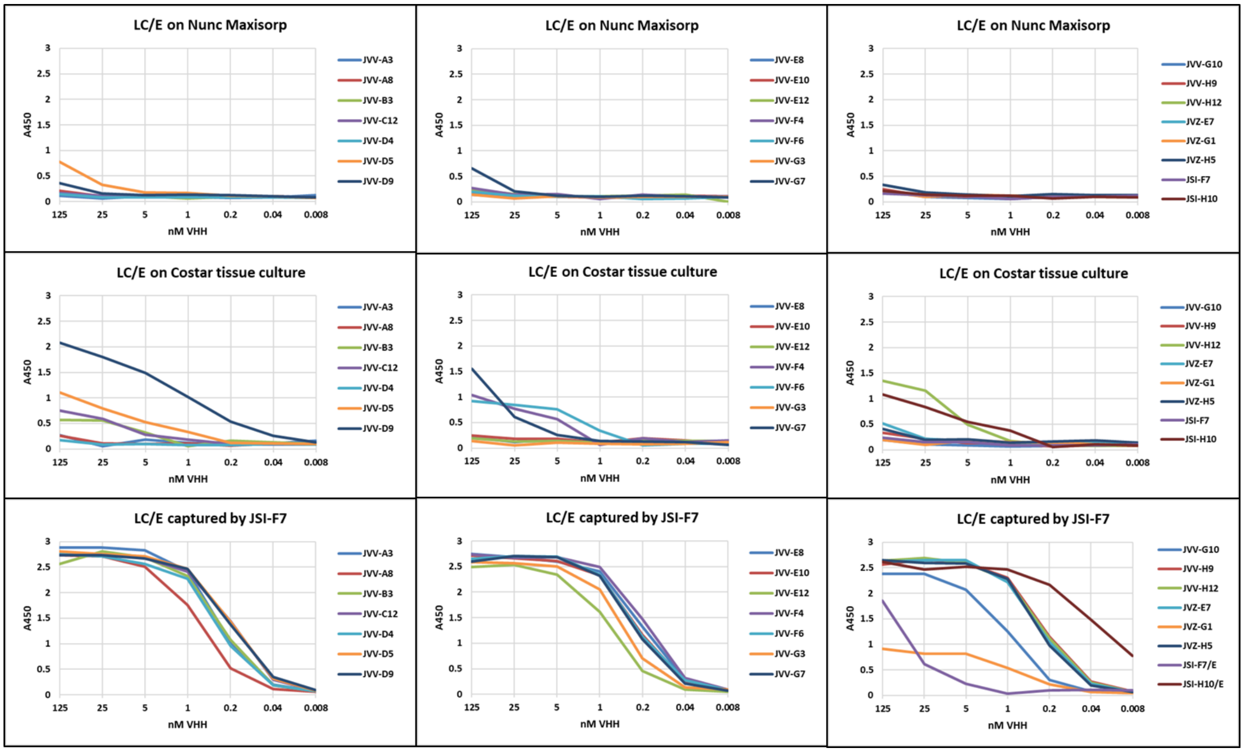

Figure S3. Dilution ELISAs of LC/E-binding VHHs on plastic coated or antibody-captured LC/E. The LC/E replicate ELISAs were performed under three conditions: 1) coating 1 µg/mL on Nunc Maxisorp; 2) coating 1 µg/mL on Costar tissue culture plastic, and; 3) pre-coating plates with 5 µg/mL JSI-H7 (expressed with myc tag), then capturing 1 µg/mL LC/E. All VHHs were diluted to 125 nM and then subjected to 1:5 serial dilutions. VHH binding was detected with HRP/anti-E-tag reagent.

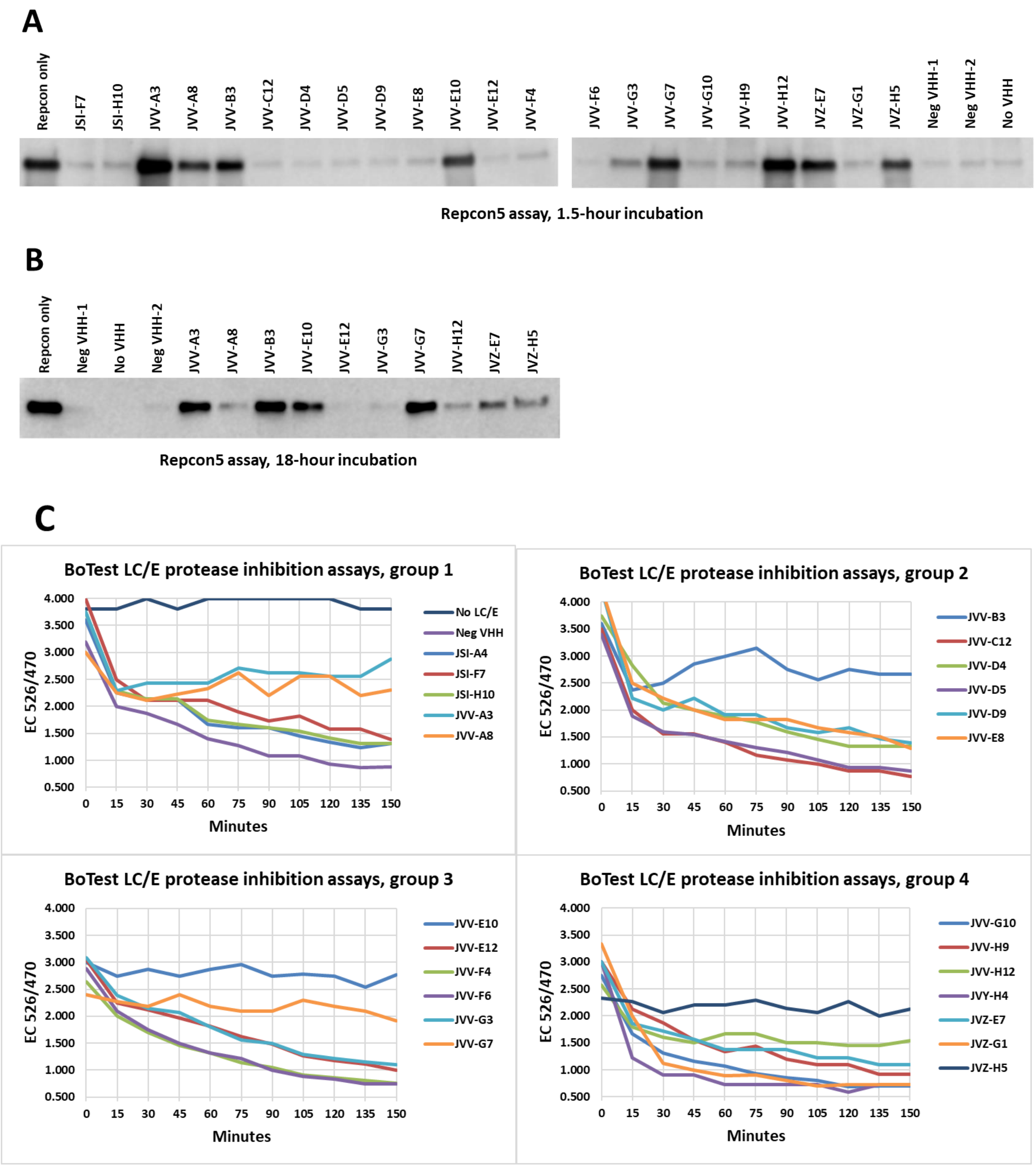

Figure S4. Screening of LC/E-binding VHHs for protease inhibition activity. For the Repcon5 Western blot assays, the indicated VHHs at 10 µg/mL were pre-incubated with 1 µg/mL of LC/E (15:1 molar ratio) prior to addition of the Repcon5 substrate. Following a 90-min (**A**) or an 18 h (**B**) incubation at 37 °C an aliquot was removed and assessed for the level of intact, uncleaved Repcon5 by Western blot. For the FRET assay (**C**), 1.6 µg/mL of indicated VHHs, 25 ng/mL of LC/E (100:1 molar ratio) and the BoTest A/E reagent were incubated for 2.5 h while monitoring the fluorescence at EC526 and EC470. LC/E cleavage of BoTest reagent results in a reduced fluorescence ratio EC526/470.

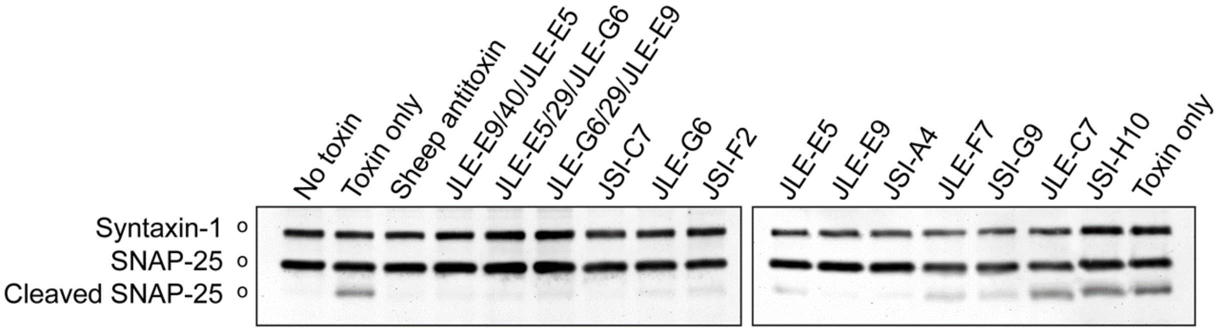

Figure S5. A subset of VHHs and VNAs have neutralizing properties in rat primary neuron cultures. Rat E18 primary cortical neuron cultures (DIV 19–23) were co-incubated with 16.7 pM BoNT/E and 200 nM each VHH or VNA for 24 h. Neuronal lysates were probed for SNAP-25 cleavage and Syntaxin-1 by immunoblot. Reduced SNAP-25 cleavage in lysates co-incubated with VNAs and a subset of VHHs indicate the ability to neutralize BoNT/E intoxication.

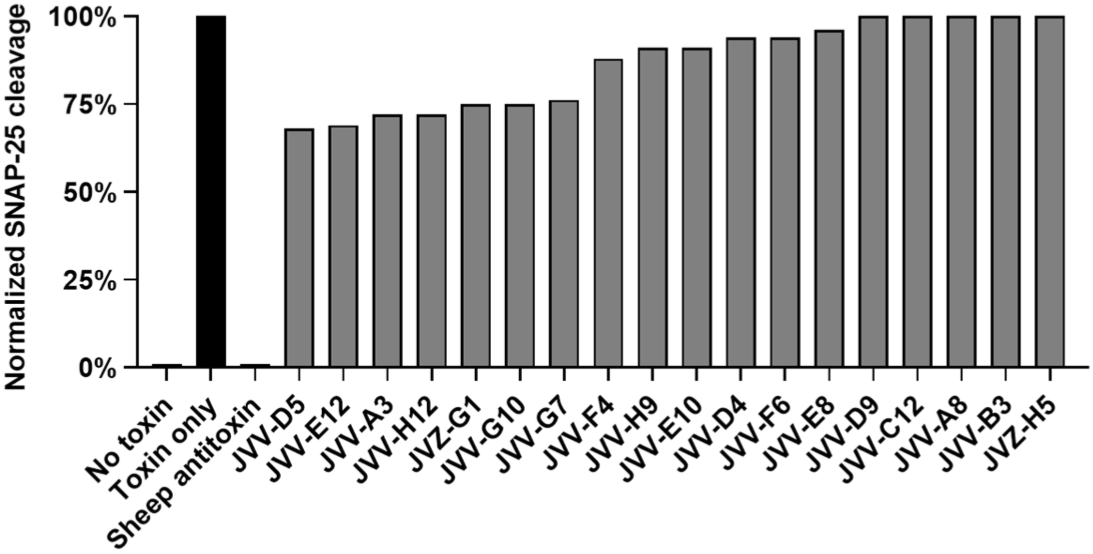

Figure S6. Summary of cell-based SNAP-25 cleavage assays for non-neutralizing VHHs. E18 primary rat cortical neurons (17–21 d in culture) were co-incubated with 200 nM each VHH and 16.7 pM BoNT/E (corresponding to 20 $MIPLD_{50}$/mL) for 24 h prior to harvest and immunoblot analysis. Listed are those VHHs that did not exhibit robust neutralizing properties compared to sheep antitoxin.

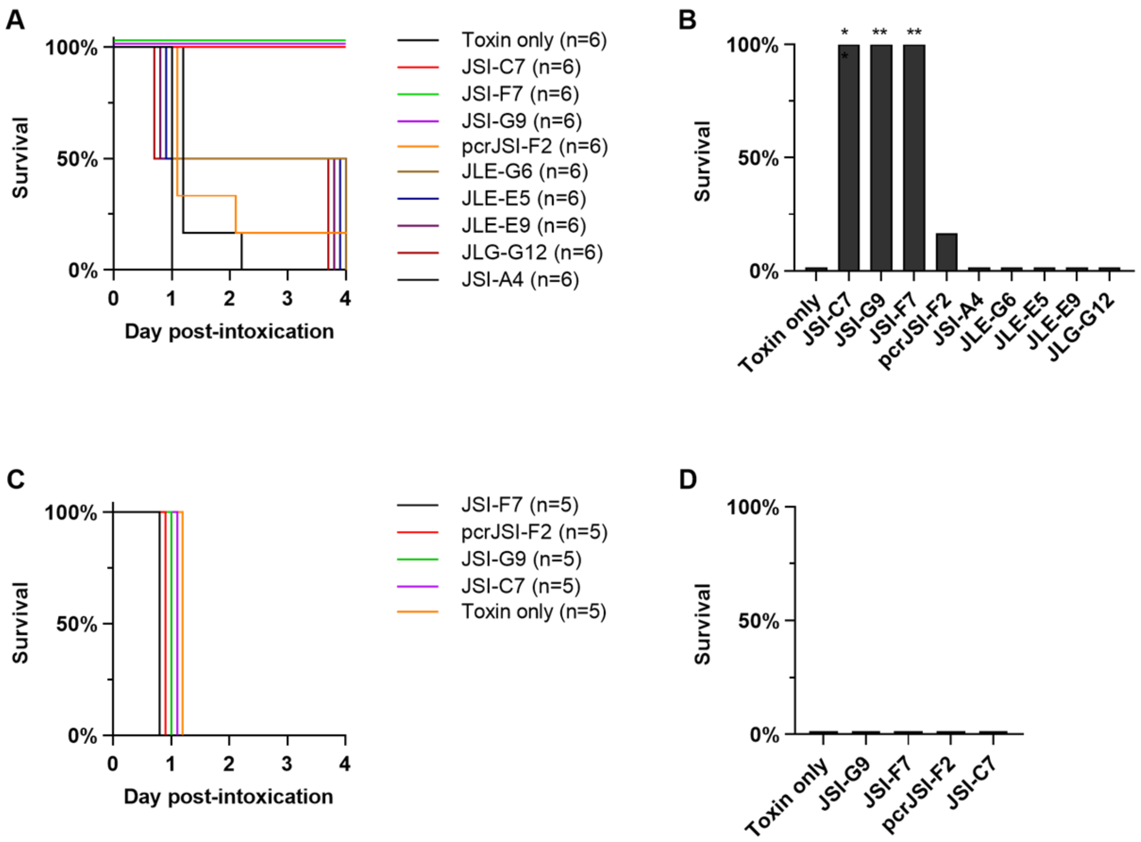

Figure S7. Select VHH monomers are able to protect mice from co-intoxication with 3 $MIPLD_{50}$ BoNT/E. Heterodimeric VNAs were prepared containing four different combinations of the three BoNT/E-neutralizing VHHs identified in Table 1. Mice (n = 6 in two replicates) were co-administered each VNA (40 pmol) with 3 (**A, B**) or 10 (**C, D**) $MIPLD_{50}$ BoNT/E and monitored for survival over 4 days. (**A**) Survival following 3 $MIPLD_{50}$ BoNT/E challenge. (**B**) Comparison of protective efficacies of each VNA at 3 $MIPLD_{50}$ BoNT/E with Bonferroni-adjusted significances against vehicle treatments. (**C**) VNAs effective at 3 $MIPLD_{50}$ BoNT/E were tested against 10 $MIPLD_{50}$ BoNT/E. (**D**) Comparison of survival proportions. ** $p < 0.01$.

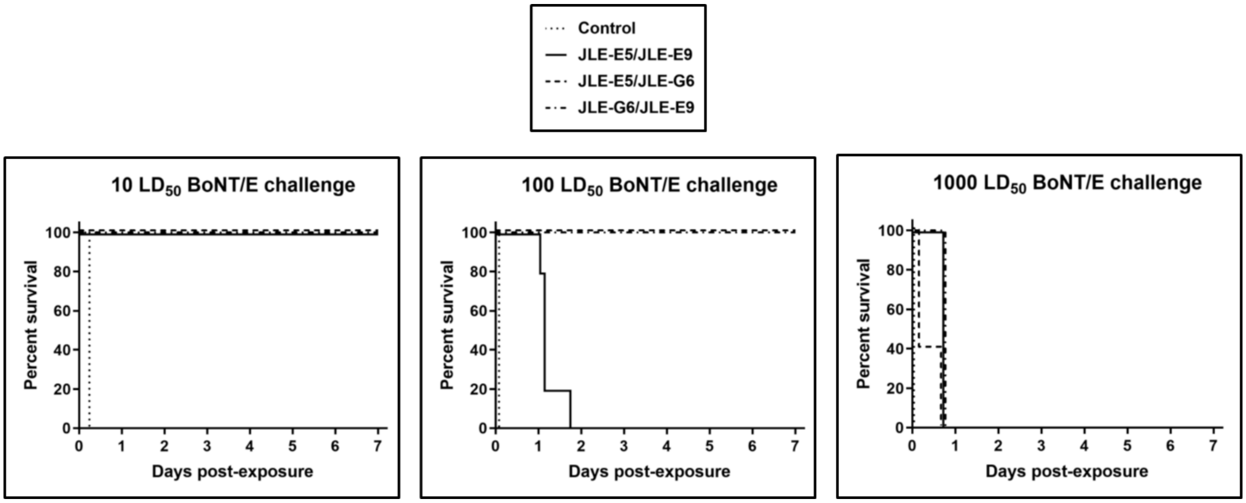

Figure S8. Second site protection study testing the BoNT/E antitoxin potency of three VHH heterodimers in mice. Heterodimeric VNAs were prepared containing three different combinations of the three BoNT/E-neutralizing VHHs identified in Table 1. Complexed botulinum neurotoxin serotype E (BoNT/E; Metabiologics, Inc.) was activated with trypsin. For this study, 100 ug trypsin (EMD) was dissolved with 83 ul HEPES (Sigma-Aldrich, St. Louis, MO) to prepare a 1.2 mg/mL solution. 50 ug of complexed BoNT/E was diluted with 25 ul 1.2 mg/mL trypsin in HEPES and 100 ul PBS and incubated for 30 min. at 37 °C. Then 25 ul of 2.5 mg/mL trypsin inhibitor (Sigma-Aldrich, St. Louis, MO) in HEPES was added and incubated 15 min. at room temperature. The resulting activated complexed BoNT/E was quantitated in vivo to determine the LD50 dose and stored at −80 °C until use. Groups of five mice were co- administered (iv) 2 μg of the indicated VNA (40 pmoles) and either 10, 100 or 1000 LD_{50} of BoNT/E (ip). Mice were then monitored several times per day for survival and signs of botulism for a week. The survival of mice is plotted as a function of time post-exposure.

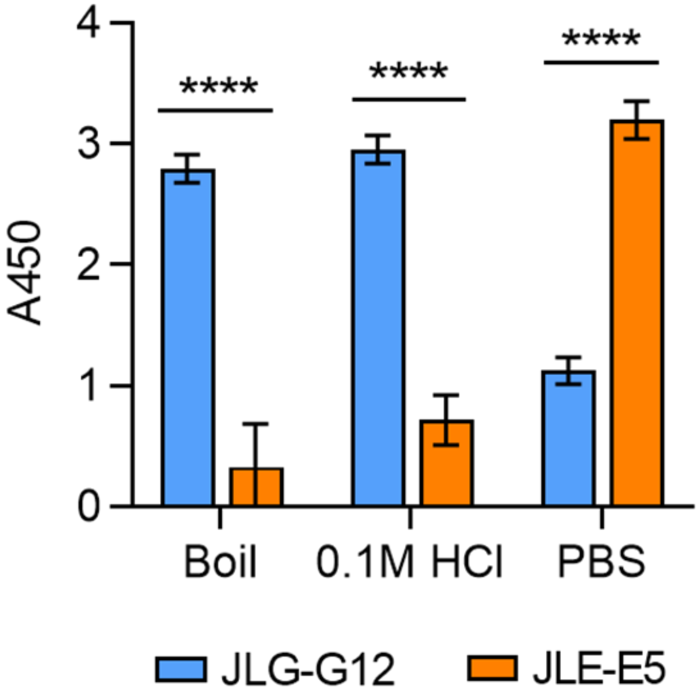

Figure S9. The JLG-G12 VHH family recognizes a stable, partially unfolded ciBoNTE conformation. A Costar tissue culture plate was coated with ciBoNTE and blocked. Wells were then pre-treated in three different ways prior to VHH binding ELISAs. In one pre-treatment, boiling PBS was quickly added to each well and allowed to cool. A second pre-treatment was to add 0.1 M HCL for 1 h. The third treatment was to incubate wells with PBS. Following pre-treatment, the wells were washed and incubated with 10 µg/mL JLG-G12 VHH or 10 µg/mL JLE-E5 VHH. The wells were then washed and bound VHH was quantified with HRP/anti-E-tag. Results shown are the average of eight replicate wells. **** $p < 0.0001$.
